# Alterations in GSH/GSSG and CyS/CySS redox status in small cell lung cancer patients undergoing chemotherapy

**DOI:** 10.1007/s12672-025-03251-2

**Published:** 2025-07-30

**Authors:** Azra Guzonjić, Milkica Crevar, Ivana Simić, Natalija Samardzić, Vesna Ćeriman Krstić, Jelena Kotur Stevuljević, Dragana Jovanović

**Affiliations:** 1 Faculty of Pharmacy, Department for Medical Biochemistry, University of Belgrade, Vojvode Stepe 450, Belgrade, 11221 Serbia; 2 Faculty of Pharmacy, Department of Pharmaceutical Chemistry, University of Belgrade, Vojvode Stepe 450, Belgrade, 11221 Serbia; 3 Merck Sharp & Dohme d.o.o, Medical Affairs, Omladinskih brigada 90a, Belgrade, 11070 Serbia; 4 Clinic for pulmonology, University Clinical Center of Serbia, dr Koste Todorovica 26, Belgrade, 11000 Serbia; 5 Faculty of Medicine, University of Belgrade, dr Subotica 8, Belgrade, 11000 Serbia; 6 Internal Medicine Clinic “Akta Medica”, Cara Nikolaja II, Belgrade, 11000 Serbia

**Keywords:** Oxidative stress, Lung cancer, Redox imbalance, Prognostic biomarkers

## Abstract

**Background:**

In small cell lung cancer (SCLC), oxidative stress disrupts redox balance and contributes to tumor progression and treatment resistance through DNA damage, inflammation, and tumorigenesis. Thiol compounds such as glutathione (GSH) and cysteine (CyS) together with their oxidized forms (GSSG and CySS) serve as markers of oxidative stress. The aim of this study was to investigate changes in GSH/GSSG and CyS/CySS ratios during chemotherapy and evaluate their potential as prognostic indicators in SCLC.

**Materials and methods:**

In this longitudinal study, redox biomarkers (GSH/GSSG and CyS/CySS ratios) were investigated in 60 stage III/IV SCLC patients receiving cisplatin-etoposide chemotherapy. Plasma samples were collected before chemotherapy, after two cycles and after four cycles and analyzed by LC-MS/MS.

**Results:**

Significant redox changes were observed during chemotherapy. The GSH/GSSG ratio decreased after two cycles (*p* = 0.029) and increased after four cycles (*p* = 0.002). The same trend was observed for CyS/CySS dynamics (*p* = 0.031 and *p* = 0.030, respectively). The Survivors showed a recovery of the redox balance, while the deceased patients showed persistently lower ratios. Kaplan-Meier analysis showed that a higher GSH/GSSG ratio before treatment (*p* = 0.037) predicted better survival. A positive correlation was found between GSH/GSSG and CyS/CySS ratios (ρ = 0.306, *p* = 0.019).

**Conclusions:**

This study demonstrates that redox imbalance reflected in GSH/GSSG and CyS/CySS ratios is an important factor for SCLC treatment response and survival. Higher GSH/GSSG ratios before treatment are associated with improved survival, indicating the potential of redox markers as prognostic tools in SCLC.

## Introduction

## Background on SCLC

Small cell lung cancer (SCLC) is an extremely aggressive malignancy that accounts for about 15% of all lung cancer cases [1]. Characterized by rapid growth and early metastatic spread, SCLC is typically diagnosed at an advanced stage (III or IV) where it is less amenable to curative surgery [2]. The cornerstone of SCLC treatment is platinum-based chemotherapy, usually the combination of cisplatin and etoposide [3]. While this treatment usually results in a strong initial response, relapse rates are exceptionally high and the overall prognosis remains poor with a five-year survival rate of less than 7% [4]. These challenges emphasize the urgent need for robust biomarkers to effectively monitor treatment response and predict clinical outcomes in SCLC patients.

### Redox status in cancer

The redox balance, which is determined by reactive oxygen species (ROS) and antioxidants, plays a pivotal role in cancer biology. Cancer cells often exhibit an altered redox status that promotes their rapid growth and survival under oxidative stress conditions [5]. Redox changes can influence important processes such as gene expression, cell cycle regulation, apoptosis and resistance to chemotherapy [6]. In addition, oxidative stress is increased in cancer cells due to the rapid proliferation and metabolic activity of tumor cells, which leads to significant changes in the redox balance. The balance between oxidative and antioxidant forces in tumor cells may also affect the susceptibility of cancer cells to chemotherapy-induced damage and thus influence treatment outcomes. The dynamics of redox status and its potential role in influencing the efficacy and resistance of chemotherapy in SCLC patients remains an active area of research.

### GSH/GSSG and cys/cyss ratios in redox biology

Glutathione (GSH) and cysteine (CyS) are of central importance for the maintenance of cellular redox homeostasis. They act as important antioxidants that neutralize reactive oxygen species and regulate the thiol-disulfide balance [7]. Disturbances in GSH and CyS levels can impair cellular defense mechanisms and thus contribute to oxidative stress and disease progression [8]. Among the various redox markers, the ratio of glutathione (GSH)/glutathione disulfide (GSSG) is one of the most studied. GSH is an important antioxidant that protects cells from oxidative damage, while GSSG is its oxidized form and serves as an indicator of oxidative stress [9]. An altered GSH/GSSG ratio is often associated with different types of cancer and may serve as a potential biomarker for treatment response and prognosis [10, 11]. Similarly, the cysteine (CyS)/cystine (CySS) ratio, which represents the balance of thiol-disulfide exchange in cells, is another important marker of redox status [12]. Both ratios are central to cellular defense mechanisms, and changes in their levels can influence chemotherapy efficacy, cellular survival and metastasis in SCLC. Monitoring these redox ratios in SCLC patients undergoing chemotherapy could provide insight into their clinical response and guide therapeutic strategies.

### Study hypotesis and objectives

This study aims to investigate changes in GSH/GSSG and CyS/CySS redox ratios in patients with SCLC undergoing platinum-based chemotherapy. Specifically, we hypothesize that chemotherapy induces significant changes in these redox markers and that these changes correlate with clinical outcome. Our aims are to: (1) to investigate the longitudinal dynamics of GSH/GSSG and CyS/CySS ratios during chemotherapy in both the SCLC cohort and survival outcome subgroups, (2) to explore the potential of these biomarkers as predictive biomarkers of clinical outcomes in SCLC patients, and (3) to evaluate the relationship between these redox markers and clinical outcomes.

## Materials and methods

### Study design and methodology

This study was conducted between October 2020 and February 2022 in a tertiary care facility, the University Hospital for Pulmonology, Clinical Center of Serbia, Belgrade. The study involved 60 eligible patients with SCLC whose disease was histologically confirmed at stage III or IV and who were candidates for platinum-based chemotherapy. All participants received the combination of cisplatin and etoposide and were examined at three time points: before the start of chemotherapy (pre-chemotherapy group), after two cycles (chemo-2 group) and after four cycles (chemo-4 group). Baseline sociodemographic data, smoking history and clinical characteristics were extracted from medical records to create a comprehensive profile for each patient. The experimental design builds on our previously study, which analyzed the associations between neutrophil-to-lymphocyte ratio (NLR), C-reactive protein (CRP), soluble programmed cell death ligand 1 (sPD-L1) and Schlafen 11 (SLFN11) with response to chemotherapy in SCLC patients [13]. In extension of this work, the current study focuses on the investigation of redox status and its dynamic changes during the course of chemotherapy in the same patient cohort. Additionally, peripheral venous blood samples were collected in 6 mL BD Vacutainer^®^ EDTA Tubes (Becton, Dickinson and Company, Franklin Lakes, NJ, USA) at the University Hospital of Pulmonology, Clinical Center of Serbia, Belgrade. Samples were immediately transported under controlled conditions at + 4 °C to the Department of Medical Biochemistry, University of Belgrade, to maintain sample integrity. Upon arrival, plasma was separated by centrifugation at 3000 rpm for 10 min using a “Univerzal Z 300” centrifuge (Hermle Labortechnik GmbH, Germany), aliquoted and stored at -80 °C until biochemical analyses were performed. Blood samples were systematically collected at three predefined time points: prior to chemotherapy initiation, after two cycles of chemotherapy, and after four cycles of chemotherapy, allowing for a detailed longitudinal assessment of redox status changes throughout the treatment course.

### Biochemical analysis

To assess oxidative stress and redox balance in SCLC patients undergoing chemotherapy, we investigated some parameters of redox status, particularly GSH/GSSG and Cys/CySS ratios, at different stages of treatment. Plasma samples were thawed and 200 µL of each sample was measured. Protein precipitation was performed by adding 300 µL of an internal standard solution (β-alanine in cold acetonitrile at a concentration of 100 ng/mL) to the plasma. The mixture was shaken for 30 s and then centrifuged at 14,000 rpm for 10 min at 4 °C. The resulting supernatant was membrane filtered and transferred to autosampler vials for analysis. Analysis was performed using a liquid chromatography system (ACELLA, Thermo Fisher Scientific Inc., Madison, WI, USA) coupled to a triple quadrupole mass spectrometer (TSQ Quantum Access MAX, Thermo Fisher Scientific Inc., Madison, WI, USA). Chromatographic separation was performed using an Acclaim™ HILIC-10 column (3 μm, 120 Å, 2.1 × 150 mm) maintained at 40 °C with the autosampler temperature set at 10 °C. The mobile phase consisted of acetonitrile and formic acid buffer (pH 3.5) in a ratio of 20:80 (v/v), with a flow rate of 500 µL/min and an injection volume of 20 µL. MS/MS analysis was performed in ESI + mode using Multiple Reaction Monitoring (MRM) for analyte detection. The monitored transitions included: m/z 241.0 → 120.2 for cystine (collision energy 1 eV), m/z 122.1 → 59.3 for cysteine (collision energy 22 eV), m/z 308.1 → 179.0 for glutathione (collision energy 7 eV), m/z 613.3 → 354.9 for oxidized glutathione (collision energy 19 eV) and m/z 90.2 → 44.6 for β-alanine (collision energy 18 eV). Optimized method parameters included a spray voltage of 5000 eV, an evaporator temperature of 250 °C, a capillary temperature of 250 °C, a sheath gas pressure of 50 arbitrary units, an auxiliary gas pressure of 20 arbitrary units, a capillary offset of 35, and a tube lens offset of 35. Data acquisition and system control were performed using TSQ Tune and XCalibur software, which ensured accurate quantification of the targeted redox markers.

### Statistical methods

The distribution of continuous variables was tested for normality using the Kolmogorov-Smirnov test for data sets with more than 50 observations and the Shapiro-Wilk test for those with fewer than 50 observations. Continuous data are presented as medians with interquartile ranges (25th– 75th percentiles), while categorical variables are presented as absolute frequencies (n) and relative percentages (%). Comparisons between SCLC groups were performed using the Friedman test, a non-parametric ANOVA for repeated measures, followed by the Wilcoxon signed-rank test for post-hoc pairwise comparisons. For subgroup analyzes within the SCLC cohort, the Kruskal-Wallis test, a non-parametric ANOVA, was applied, with post-hoc comparisons performed using the Mann-Whitney U test. Differences in categorical variables were analyzed using the Chi-square test. Spearman’s rank correlation coefficient was used to examine the relationships between measured parameters, while Kaplan-Meier survival analysis was used to estimate survival functions based on the risk values of the analyzed parameters. All statistical analyzes were performed using IBM^®^ SPSS^®^ Statistics software, version 20 (IBM Corporation, Chicago, IL, USA), with a significance threshold of *p* < 0.05.

## Results

### Overview of the study population

In this study, 60 patients with histologically confirmed SCLC were examined and divided into two groups based on their survival status: survivors (*n* = 25) and deceased (*n* = 35). Sociodemographic characteristics such as age, gender, smoking status and clinical variables such as tumor stage and comorbidities were compared between the groups (Table 1). No statistically significant differences in baseline parameters were found (*p* > 0.05), ensuring comparability of the groups for subsequent analyzes. This homogeneity provides a solid basis for the assessment of redox status as a potential biomarker for prognosis and response to treatment.

**Table 1:** Characteristics of SCLC patients by survival outcomes: sociodemographic, smoking, and clinical factors

Variable	SURVIVORS *n* = 25	DECEASED *n* = 35	*p*
Age (years)	66 (61–70)	67 (61–72)	0.775
Gender (m/f)	17 (68)/8 (32)	20 (57)/15 (43)	0.394
Cigarette smoking (yes/no)	19 (76)/6 (24)	24 (69)/11 (31)	0.625
Pack-years categories light smoking (< 10)/moderate smoking (10–19)/heavy smoking ($\:\ge\:$20)	1 (4)/0 (0)/24 (96)	0 (0)/0 (0)/35 (100)	0.240
Cardiovascular diseases (yes/no)	9 (36)/16 (64)	18 (51)/17 (49)	0.236
Primary tumor size (T) 0/1/2/3/4	2 (2)/2 (1)/4 (6)/6 (6)/11(20)	2 (6)/1(3)/6 (17)/6(17)/20(57)	0.786
Regional lymph nodes (N) 0/1/2/3	4 (16)/3 (12)/12 (48)/6(24)	5 (14)/3 (9)/19 (54)/8(23)	0.956
Distant metastasis (M) 0/1a/1b/1c	12 (48)/4 (16)/3 (12)/6(24)	20 (57)/4(11)/3 (9)/8(23)	0.888
Limited stage/Extensive stage	12 (48)/13 (52)	20 (57)/15 (43)	0.484

Categorical data are presented as proportions (n/%) and numerical data as medians with interquartile ranges, with comparisons using the chi-square (χ²) test for categorical variables and the mann-whitney u test for numerical variables. *p* < 0.05 was considered statistically significant. sclc - small cell lung cancer

### Redox status in SCLC patients

**Fig. 1:**
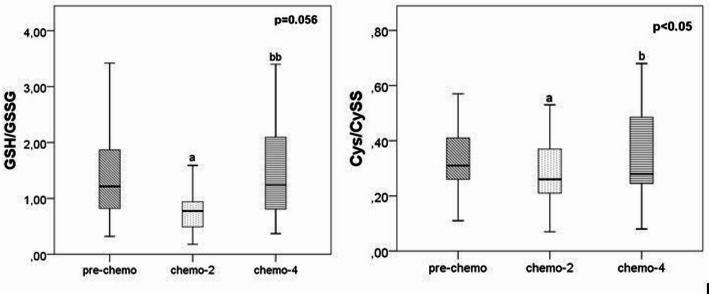
Distribution of GSH/GSSG and CyS/CySS in SCLC patients. Data were compared using the Friedman (p value) + post-hoc Wilcoxon test (^a, aa^ *p* < 0.05; 0.01 vs. pre-chemo; ^b, bb^ *p* < 0.05; 0.01 vs. chemo-2). P < 0.05 was considered statistically significant. SCLC - small cell lung cancer

Dynamic changes in the redox status parameters GSH/GSSG and CyS/CySS were observed during the entire treatment period. After the start of chemotherapy, a significant decrease in both ratios was observed, as shown in Fig. 1 The GSH/GSSG ratio decreased significantly compared to baseline after two cycles (*p* = 0.029) and then increased significantly after four cycles (*p* = 0.002). Similarly, CyS/CySS ratios showed a progressive decrease during the first two cycles of chemotherapy (*p* = 0.031).

### Redox status in survival outcome subgroups

**Fig. 2:**
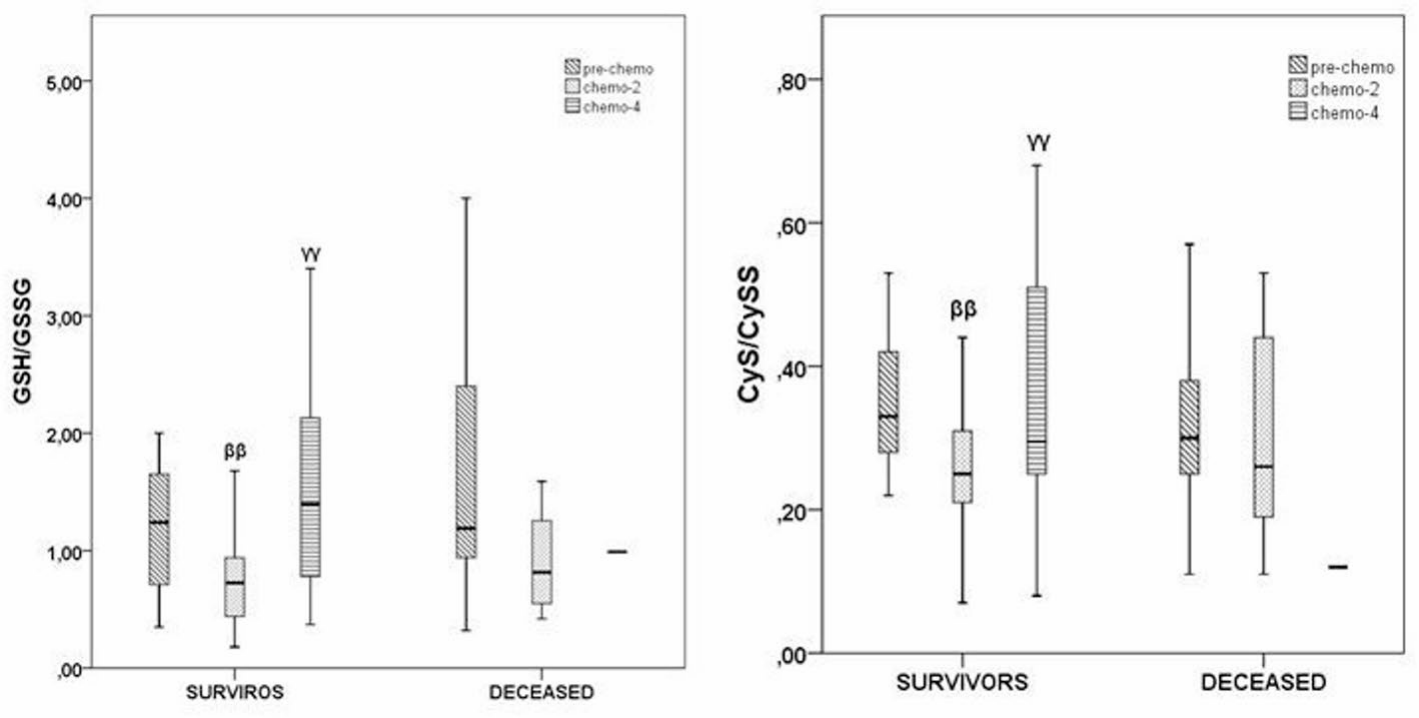
Distribution of GSH/GSSG and CyS/CySS ratios across survival outcome subgroups. Data were compared using the Friedman (p value) + post-hoc Wilcoxon test (^β, ββ, βββ^ *p* < 0.05; 0.01; 0.001 vs. pre-chemo survivors; ^*γ, γγ, γγγ*^ *p* < 0.05; 0.01; 0.001 vs. chemo-2 survivors). *P* < 0.05 was considered statistically significant

A comparative analysis of redox status between the subgroups of survivors revealed different trends in redox dynamics during chemotherapy. In survivors, a significant and sustained decrease in GSH/GSSG and CyS/CySS ratios was observed after two cycles of chemotherapy, followed by a remarkable recovery of both parameters (*p* = 0.005 and *p* = 0.004 for GSH/GSSG; *p* = 0.008 and *p* = 0.015 for CyS/CySS) (Fig. 2). In contrast, the deceased patients had a relatively stable but overall lower redox profile, with GSH/GSSG and CyS/CySS ratios decreasing only slightly throughout treatment, although a similar initial decrease was observed after two cycles. The consistently lower levels of both parameters in deceased patients highlight the inability to maintain redox homeostasis, suggesting that a disturbed redox balance may contribute to poorer clinical outcomes.

### Survival analysis of redox status parameters

**Fig. 3:**
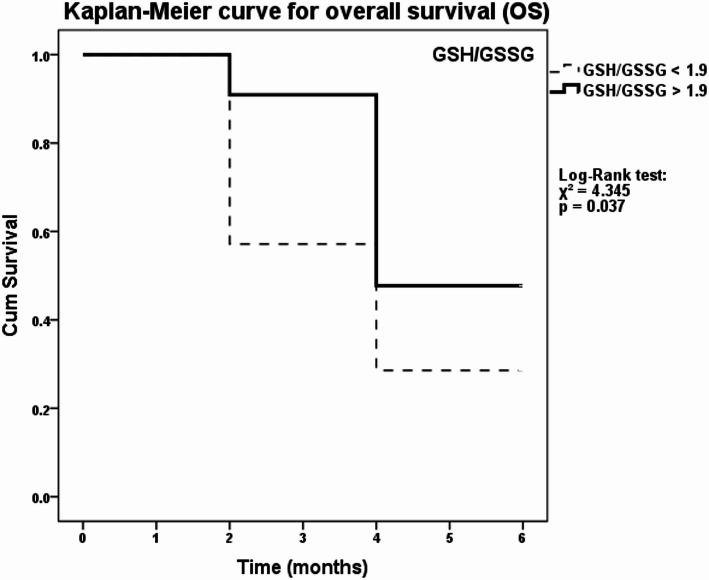
Kaplan-Meier survival analysis of GSH/GSSG ratio in SCLC patients

The Kaplan-Meier survival analysis showed the prognostic significance of the GSH/GSSG ratio (Fig. 3). Patients with values above the 75th percentile of the GSH/GSSG ratio (GSH/GSSG > 1.9) before chemotherapy had a significantly longer overall survival (OS) than patients with a lower ratio (Log-Rank test: χ^2^ = 4.345, *p* = 0.037). Additionally, analysis of progression-free survival (PFS) revealed a similar pattern, with significantly longer PFS in the high GSH/GSSG group (Log-Rank test: χ^2^ = 5.793, *p* = 0.016). These findings reinforce the potential of the GSH/GSSG ratio as a prognostic biomarker in SCLC. However, the analysis did not reveal any significant association between the CyS/CySS ratio and overall survival. The GSH/GSSG ratio proves to be a promising biomarker for the stratification of SCLC patients and the adaptation of therapeutic approaches. In addition to Kaplan-Meier analysis, univariate Cox regression was performed to evaluate the prognostic significance of GSH/GSSG and CyS/CySS ratios, as well as clinical variables including age, sex, smoking status, and disease stage. However, none of the tested variables showed statistically significant associations with overall survival (*p* > 0.05). Therefore, multivariate analysis was not conducted.

### Correlations of redox status parameters

**Fig. 4:**
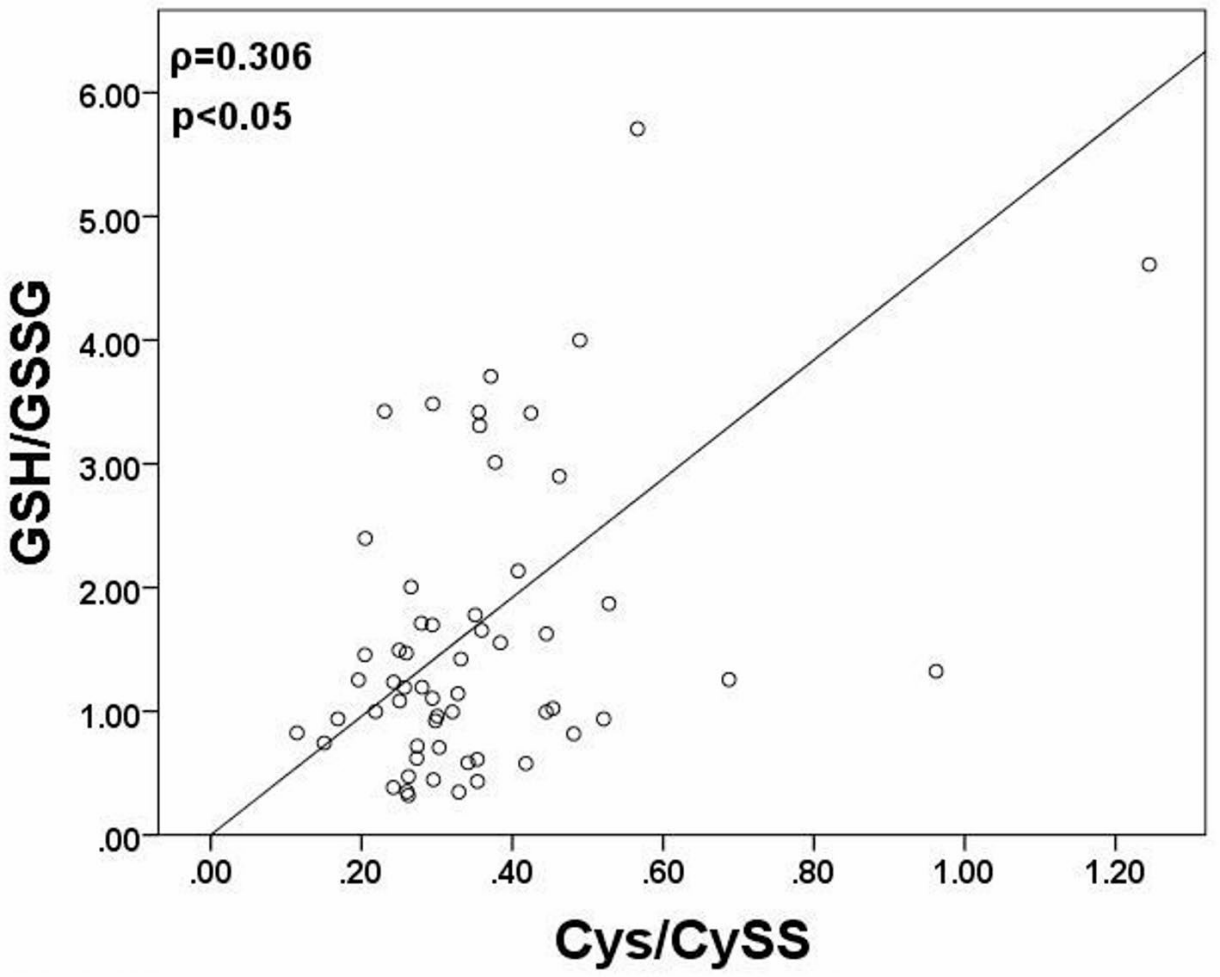
Spearman’s correlation analysis in pre-chemo group

Spearman’s rank correlation analysis showed a significant positive relationship between the redox status parameters in the pre-chemotherapy group (Fig. 4). Higher GSH/GSSG ratios were significantly associated with higher CyS/CySS ratios (ρ = 0.306, *p* = 0.019), indicating a coordinated regulation of intracellular and extracellular redox balance.

### Redox status across subgroups

**Fig. 5:**
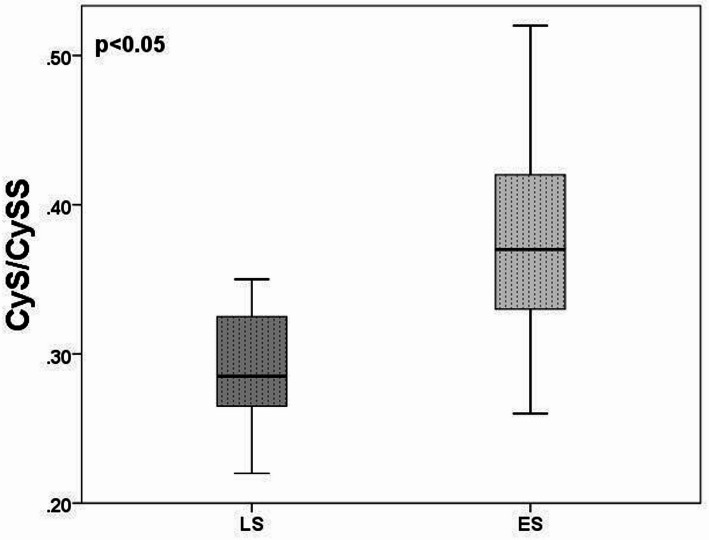
Comparison of CyS/CySS ratio by LS/ES status in pre-chemo SCLC survivors. Data were compared using the Mann-Whitney U test (p-value). *P* < 0.05 was considered statistically significant. SCLC - small cell lung cancer, LS - limited stage, ES - extensive stage

A subgroup analysis based on disease stage (limited vs. extensive) revealed significant differences in CyS/CySS ratios at baseline. Patients with extensive disease (ES) had higher CyS/CySS ratios compared to patients with limited disease (LS) (*p* = 0.044, Fig. 5). No further significant differences were found in a subgroup of patients according to other clinical parameters.

**Fig. 6:**
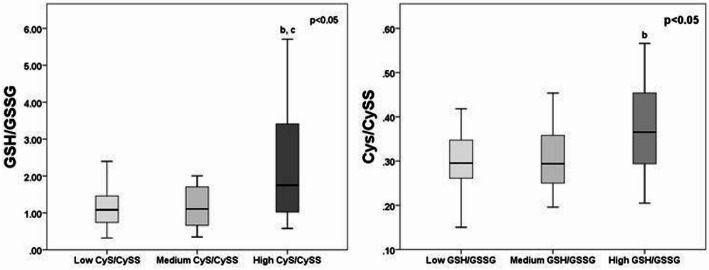
Comparative analysis of GSH/GSSG and CyS/CySS across tercile-based subgroups in pre-chemo SCLC patients. Data were compared using the Kruskal Wallis (p-value) + post-hoc Mann-Whitney U test (^b, bb, bbb^ *p* < 0.05; 0.01; 0.001 vs. low-ratio group; ^c, cc, ccc^ *p* < 0.05; 0.01; 0.001 vs. medium-ratio group); *P* < 0.05 was considered statistically significant. SCLC - small cell lung cancer

In addition, tercile-based stratification of GSH/GSSG and CyS/CySS ratios in the pre-chemotherapy cohort provided deeper insight into the relationship between these two parameters (Fig. 6). Patients with the highest CyS/CySS ratios at baseline had significantly higher GSH/GSSG ratios, highlighting a strong interdependency between these redox markers at baseline.

## Discussion

The results of this study provide new insights into the role of redox homeostasis in SCLC patients undergoing chemotherapy. While there is a lack of comprehensive studies directly investigating redox dynamics in SCLC during chemotherapy, our study provides valuable insight into the changes in GSH/GSSG and CyS/CySS ratios during treatment. To our knowledge, this is one of the first studies to investigate these highly significant biomarkers of redox balance in SCLC patients, providing a new perspective on redox homeostasis and response to chemotherapy. Our results show that the redox homeostasis of SCLC patients fluctuates during chemotherapy and that these changes are associated with patient survival. The correlation between GSH/GSSG and CyS/CySS ratios emphasizes the idea of coordinated regulation of redox balance.

An important finding of our study is the significant correlation between the pre-treatment GSH/GSSG ratio and patient survival, which emphasizes the role of a balanced redox environment in increasing treatment efficacy and improving patient survival. Patients with a higher baseline GSH/GSSG ratio (above the 75th percentile) had significantly longer overall survival, emphasizing the prognostic value of this redox marker. This finding is consistent with previous research showing that a balanced redox environment is critical for improving treatment efficacy and survival in cancer patients [14]. Other studies suggest that the GSSG/GSH ratio has the potential of a promising and simple assay that could be further validated as a novel clinical biomarker for cancer diagnosis and monitoring [15, 16]. This is consistent with our hypothesis that the GSSG/GSH ratio could play a similar role in cancer detection and treatment. However, the association observed in the Kaplan-Meier analysis was not confirmed in the Cox regression model. This discrepancy may be due to the limited sample size, reduced statistical power, or differences in how the two methods handle time-to-event data. Further studies with larger cohorts are warranted to validate these findings.

Furthermore, these results raise hypotheses about the tumor microenvironment in SCLC. It is plausible that tumors grow in an atmosphere of high oxidative stress, in which tumor cells could survive by accumulating antioxidants to neutralize the deleterious effects of oxidative stress [17]. Alternatively, the oxidative stress induced by chemotherapy could increase ROS levels to a threshold that exceeds cell survival mechanisms and leads to tumor cell death. In patients with poorer outcomes, it is possible that their tumors accumulate antioxidants more efficiently, thereby neutralizing the effects of chemotherapy-induced oxidative stress.

In this study, we observed significant dynamic changes in redox parameters during chemotherapy. Both GSH/GSSG and CyS/CySS ratios showed a significant decrease after two cycles of chemotherapy, which is consistent with the induction of oxidative stress by chemotherapeutic agents and indicates cumulative oxidative stress during treatment. These results emphasize the marked disruption of redox homeostasis in SCLC patients undergoing chemotherapy. Chemotherapy, especially with agents such as cisplatin and etoposide, is known to induce the production of ROS, which significantly disrupt redox homeostasis [18]. Cisplatin exerts its cytotoxic effect primarily through DNA damage, which triggers cellular oxidative stress through the formation of ROS, especially hydroxyl radicals and hydrogen peroxide [19]. This in turn leads to a depletion of GSH, an important antioxidant that mitigates cellular oxidative damage. A reduction in GSH levels leads to an imbalance between oxidative and antioxidant processes, which further exacerbates cellular stress [20]. Similarly, etoposide, a chemotherapeutic agent used to treat SCLC, generates ROS by stabilizing the topoisomerase II-DNA complex, leading to the formation of DNA breaks and oxidative damage [21]. This also disrupts the GSH/GSSG balance, which promotes the accumulation of oxidized glutathione (GSSG) and increases oxidative stress. In addition, CyS levels are significantly disrupted by chemotherapy-induced oxidative stress, as cisplatin and etoposide promote the formation of ROS, leading to the oxidation of reduced cysteine to cystine and increased consumption of CyS for glutathione synthesis, which ultimately disrupts the CyS/CySS ratio and contributes to a pro-oxidant cellular environment [22]. Our results suggest that the observed decrease of GSH and CyS in the blood may also reflect their redistribution towards the tumors. This could support the hypothesis that in patients with poorer outcomes, antioxidants were preferentially consumed in tumor tissue, decreasing their systemic levels. Such mechanisms could explain the lower GSH/GSSG and CyS/CySS ratios in the blood of these patients. This disruption of the redox balance is thought to contribute to the poor prognosis and treatment resistance observed in SCLC patients undergoing chemotherapy. The GSH/GSSG ratio recovered significantly after four cycles of chemotherapy, indicating the potential for adaptive antioxidant responses in some patients. This recovery may reflect not only the activation of defense mechanisms against ROS, as previously reported in other studies on chemotherapy-induced oxidative stress [23], but also a reduced disease burden due to effective therapeutic response. As oxidative stress has been shown to correlate with tumor burden [15], the normalization of redox ratios in the survivor group may be at least partially attributed to tumor shrinkage during treatment. This possibility should be considered as a limitation in the interpretation of redox dynamics. Similarly, CyS/CySS ratios showed the same recovery trend, suggesting that extracellular redox changes are coordinated with intracellular ones. These results suggest that monitoring both intracellular and extracellular redox markers during chemotherapy could provide valuable insights into patient response and help determine treatment strategies.

In contrast, the patients who died in the first six months of the study had a more stable but consistently lower redox profile, particularly in the GSH/GSSG and CyS/CySS ratios. This pattern suggests that an inability to restore redox balance during chemotherapy may contribute to treatment resistance and poor clinical outcomes. Our study further suggests that maintaining or restoring redox homeostasis during treatment may be crucial for improving outcomes in SCLC patients.

A significant positive correlation was observed between the baseline ratios of GSH/GSSG and CyS/CySS. This correlation is consistent with the expected relationship between these two markers of redox balance. Since both the GSH/GSSG and CyS/CySS ratios reflect the balance between reduced and oxidized thiols, it is plausible that changes in one ratio would affect the other. Higher GSH/GSSG and CyS/CySS ratios indicate a more favorable redox environment and lower oxidative stress. The positive correlation observed in our study supports the idea that both intracellular and extracellular redox status are tightly regulated and that this coordinated regulation is critical for the maintenance of overall redox homeostasis. Furthermore, the finding that the GSH/GSSG ratio is significantly associated with survival outcome suggests that a balanced redox milieu, as indicated by intracellular and extracellular markers, plays an important role in treatment efficacy and prognosis in SCLC.

We also investigated the relationship between redox markers and disease stage. Our subgroup analysis revealed that patients with extensive stage (ES) SCLC had a significantly higher CyS/CySS ratio at baseline than patients with limited stage (LS) disease. Since a higher CyS/CySS ratio indicates a more reduced extracellular redox state, this finding may at first seem contradictory to previous reports associating advanced disease with increased oxidative stress [15, 24]. However, it is possible that the CyS/CySS ratio reflects a compensatory extracellular redox response, rather than directly mirroring intracellular oxidative burden. This may suggest a more complex, compartment-specific redox regulation in SCLC. Alternatively, these findings could indicate that redox remodeling in advanced SCLC involves not only enhanced oxidative stress but also altered antioxidant responses, particularly in the extracellular environment. Therefore, the CyS/CySS ratio might still serve as a prognostic biomarker, reflecting disease-associated redox dynamics beyond classical oxidative stress paradigms.

While this study provides valuable insights into the role of redox status in SCLC, there are several limitations that should be addressed in future research. First, the sample size was relatively small, especially in the subgroup analysis, which may limit the generalizability of our findings. Future studies with larger cohorts are needed to validate these results and further explore the mechanisms by which redox status influences chemotherapy response and survival in SCLC. Furthermore, exploring the relationship between redox markers and specific chemotherapeutic agents could help optimize treatment strategies for individual patients. Additionally, the absence of paired plasma and tumor tissue samples in this study limits the direct interpretation of systemic biomarkers as a precise reflection of intratumoral conditions.

## Conclusions

In conclusion, our study highlights the importance of redox status, as reflected in GSH/GSSG and CyS/CySS ratios, as a potential biomarker for prognosis and response to treatment in SCLC patients undergoing chemotherapy. This area remains under-researched, with few studies investigating the dynamic changes of these redox markers during treatment. Our findings highlight the novelty and potential importance of monitoring these ratios, as their association with survival outcomes suggests that maintaining redox homeostasis may be critical for improving treatment efficacy and patient survival. The observed positive correlation between GSH/GSSG and CyS/CySS ratios supports the concept of coordinated regulation of intracellular and extracellular redox balance. These results improve our understanding of oxidative stress in SCLC and highlight the potential value of redox biomarkers in stratifying patients for personalized treatment strategies. The dynamic shifts in these ratios during treatment offer actionable insights, particularly in cancers such as SCLC where oxidative stress plays a central role in therapy resistance and tumor aggressiveness. The GSH/GSSG and CyS/CySS ratios therefore hold considerable clinical potential as redox biomarkers in oncology, offering a more specific and actionable approach to patient management in this under-researched area of research. However, it remains uncertain whether these systemic redox alterations fully represent the intratumoral oxidative stress profile, which may differ due to localized redox regulatory mechanisms and tumor heterogeneity.

## Data Availability

The datasets generated during and/or analyzed during the current study are available from the corresponding author upon reasonable request.
